# Retraction Note: Who performs the preoperative examination?

**DOI:** 10.1186/s40981-020-00347-2

**Published:** 2020-06-03

**Authors:** Hironobu Ueshima, Hiroshi Otake

**Affiliations:** Department of Anesthesiology, Showa University Hospital, Tokyo, Japan

**Retraction Note: JA Clin Rep (2018) 4:21**

**https://doi.org/10.1186/s40981-018-0161-6**

The Editor-in-Chief has retracted this article [1]. Following an investigation by the editorial board, it was found that the information presented in the Letter to the Editor is not reliable because the clinical pathway of the patient was not accurately reported.

All authors agree to this retraction.
